# CT radiomics-histopathological correlation for mediastinal lymph node staging in non-small cell lung cancer

**DOI:** 10.1371/journal.pone.0355650

**Published:** 2026-08-31

**Authors:** Judith Legrand, Antoine Decoux, Loïc Duron, Cécile Di-Rocco, Manuel Gargiulo, Pascal Thomas, Armelle Arnoux, Kathia Chaumoitre, Jean-Yves Gaubert, Laure Fournier, Paul Habert

**Affiliations:** 1 Imaging Department, Hopital Nord, APHM, Aix Marseille University, France; 2 Université Paris Cité, PARCC UMRS, INSERM, Paris, France; 3 Department of Neuroradiology, Alphonse de Rothschild Foundation Hospital, Paris, France; 4 Aix Marseille Université, LIIE, Marseille, France; 5 Department of Thoracic Surgery and Lung Transplantation, hôpital Nord, chemin des Bourrely, Marseille, France; 6 Aix Marseille Université, Marseille, France; 7 Université Paris Cité, AP-HP, Hopital européen Georges Pompidou, Unité de Recherche Clinique, Centre d’Investigation Clinique Épidémiologie Clinique, INSERM, Paris, France; 8 Department of Radiology, AP-HM, Hôpital La Timone, Marseille, France; 9 Université Paris Cité, AP-HP, Hopital européen Georges Pompidou, PARCC UMRS, INSERM, Paris, France; University of Pisa, ITALY

## Abstract

**Background:**

Non-small cell lung cancer (NSCLC) staging relies on accurate assessment of mediastinal lymph nodes. This study investigates the utility of radiomic features derived from contrast-enhanced thoracic CT scans in predicting malignancy in clinically positive (cN+) mediastinal lymph nodes.

**Methods:**

A retrospective cohort of 110 NSCLC patients with cN + who underwent surgical resection was analyzed. 3D segmentations of up to three lymph nodes per patient were performed. Radiomic features, encompassing heterogeneity measures, were extracted. A radiomics model was constructed using a random forest and XGboost algorithm. To ensure robustness and minimize bias, 100 iterations of data splitting were conducted to create distinct training and test sets for reproducibility and statistical reliability.

**Results:**

The radiomics model achieved an area under the curve (AUC) of 0.703 for Forest model and 063 for XGboost model. Three recurrent features in the radiomic signatures, “RootMeanSquared”, “Grey Level Co-Occurrence Matrix Imc2”, and “Grey Level Run Length Matrix RunEntropy”, highlighted the importance of nodal heterogeneity features in the model.

**Conclusion:**

Radiomic features extracted from contrast-enhanced thoracic CT scans could predict malignancy in cN+ mediastinal lymph nodes of NSCLC patients (AUC = 0.703). Three features of heterogeneity were recurrent in radiomic signature, emphasizing the potential importance of incorporating nodal heterogeneity criteria in addition to size assessment.

## Background

Non-small cell lung cancer (NSCLC) remains a frequent disease with a poor prognosis, with over 130,000 deaths in the USA in 2021. The best survival rates occur when the disease is limited to the thorax and when complete surgical removal and lymph node dissection are performed. Mediastinal lymph node involvement is one of the leading prognostic factors. Five-year overall survival after surgery is 59.8% for patients without lymph node metastasis, and 32.9% for patients with mediastinal lymph node metastasis [1].

Imaging helps determine the extent of the disease. The eighth edition of the Tumor-Node-Metastasis (TNM) classification is the most recent for lung cancer [2]. Clinically abnormal lymph nodes (cN+) is a merged criterion based on chest CT and PET-CT results [3]. The diagnostic performance of chest CT alone for classifying nodal involvement remains limited, with a pooled sensitivity of 0.61 and a specificity of 0.79 [4]. The combination of PET/CT and CT yields better results for mediastinal staging than PET/CT or CT alone [5]. The differentiation between cN1, cN2, and cN3 depends on anatomical localization relative to the primary tumor site. The N classification of TNM is essential for determining the treatment, especially for cN2 or cN3 cases [6]. Currently, this combination lacks sensitivity, requiring histological confirmation of all cN+ lymph nodes on imaging, especially to prove the stage III disease [7,8]. Lymph node staging can be performed by endobronchial ultrasound or mediastinoscopy [9–11], which allows confirmation of imaging-positive findings.

Radiomics analysis is a data-driven research field involving the high-throughput extraction of quantitative features from medical images. It is designed to discover new imaging biomarkers and enable phenotypic profiling of lesions, with increasing interest in personalized medicine, especially in oncology [12] and lung cancer [13]. Radiomics analysis using machine learning methods has demonstrated high diagnostic performance in predicting outcomes in chest diseases and other areas of medical imaging [14–16].

The aim of this study was to create a radiomic feature-based signature to predict the malignant status of cN+ mediastinal lymph nodes on contrast-enhanced chest CT, based on 3D segmentations.

## Methods

### Population

This monocentric retrospective cohort study was conducted from January 2010 to January 2021. The institutional review board approved the study (Comité d’Ethique pour la Recherche en Imagerie Médicale n°CRM-2112–220). From a database of thoracic surgeons, all patients who underwent surgery for NSCLC with lymphnode removal, without neoadjuvant chemotherapy from 2010 to 2021 were analyzed.

Inclusion criteria were:

1) mediastinal lymph node classified as cN+ prior to surgery by a multidisciplinary team. cN + was defined as a lymph node short axis ≥ 10 mm on CT scan and/or a maximum standardized uptake value (SUV_max_) ≥ 2.5 on PET-CT [3];
2) and surgical resection of lung cancer with a histopathological report including complete thoracic lymphadenectomy, specifying the anatomical location and the metastatic or inflammatory status of each lymph node.

Exclusion criteria were:

1) absence of a chest CT scan or availability of unenhanced CT only;
2) poor image quality precluding lymph node segmentation;
3) time interval between CT and surgery > 3 months.

### Chest CT

CT scan data were collected from 01/03/2022–01/06/2022. Patient data were processed anonymously.

CT scans were performed using various systems which are detailed in supplementary material (Table e1). Contrast injection was performed via peripheral venous access. The patients were positioned supine, with arms raised, and instructed to hold their breath during acquisition after brief training with technicians. The final histological type, tumor localization, and its maximum size were recorded.

### Lymph node selection and segmentation

Up to three cN+ lymph nodes were selected for each patient and labeled according to their anatomical station number location on CT scan and their histological status (benign or malignant) based on the lymphadenectomy histopathological report.

**Fig 1:**
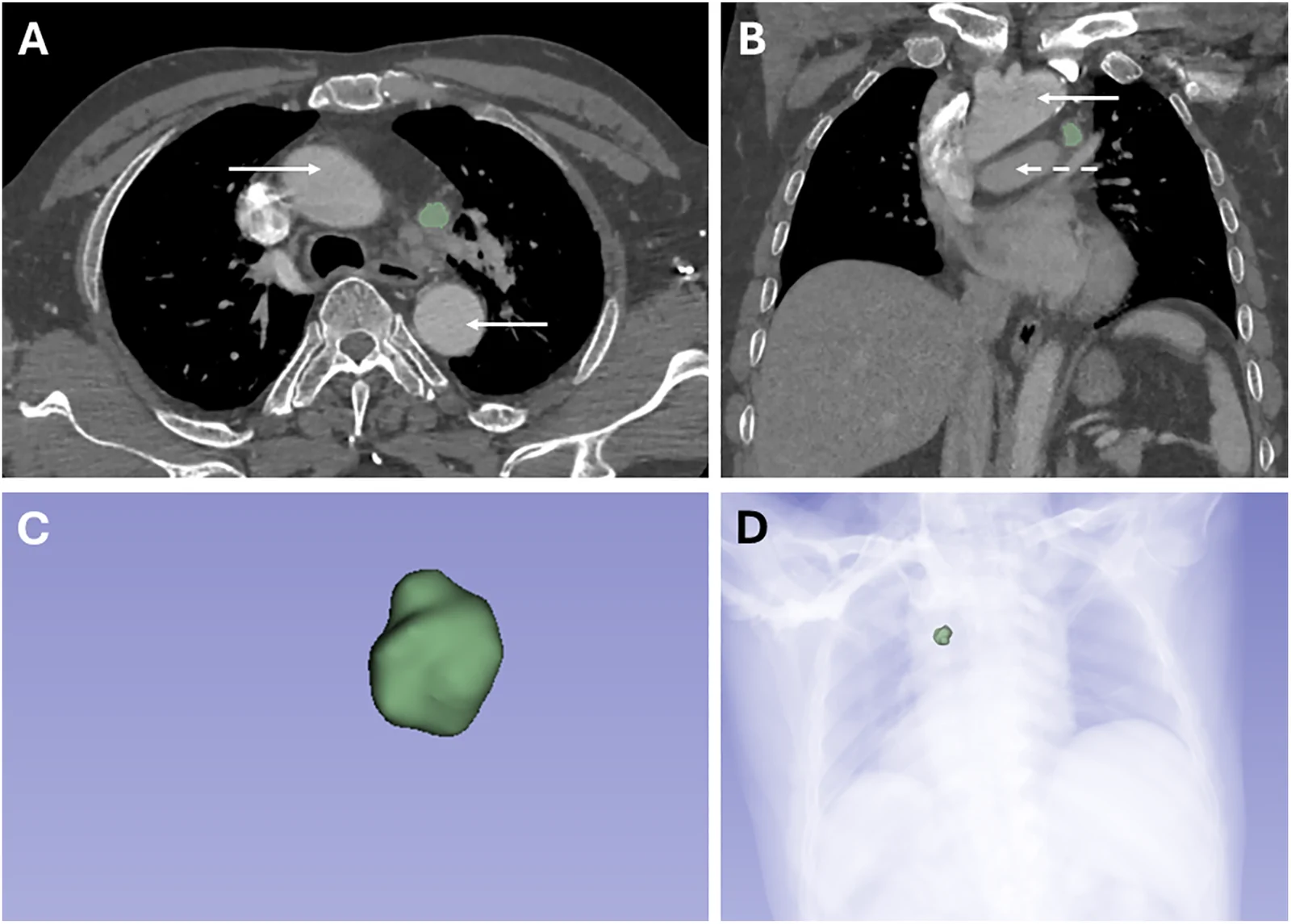
Example of lymph node segmentation. A and B. Axial (A) and frontal (B) sections showing segmentation (in green) of a lymph node in the subaortic lymphatic area (5) (aorta: solid arrow; left pulmonary artery: dashed arrow). C. 3D view of the segmented mediastinal lymph node. D. 3D view of the segmented mediastinal lymph node within the thorax.

Native/raw DICOM files from contrast-enhanced chest CT scans were extracted from the institutional PACS. Segmentations were performed using 3D Slicer (open-sourced software, www.slicer.org). Two radiologists, blinded to each other’s segmentations and histopathological results, performed the manual segmentations: one chest radiologist (P.H.) with more than 8 years of experience and one radiology resident (J.L.) with 4 years of experience. The mediastinal lymph nodes were segmented in 3D using a spherical brush tool. Up to three lymph nodes per patient were segmented. Additionally, as in clinical practice, a 2D measurement of the short axis of each lymph node was performed on the axial slice where the node appeared largest (Fig 1).

### Feature extraction and selection

For each lymph node, a total of 99 radiomic features were extracted; 10 morphological features, 18 first-order histogram features, and 71 second-order texture features (22 from the Grey Level Co-Occurrence Matrix; 14 from the Grey Level Dependance Matrix; 16 from the Grey Level Run Length Matrix; 16 from the Grey Level Size Zone Matrix; 5 from the Neighborhood Grey Tone Difference Matrix). The methodology for the extraction and analysis of radiomic features was performed in accordance with the Image Biomarker Standardization Initiative guidelines [17] (Fig 3).

### Selection of features and construction of radiomics model

Lymph nodes were randomly divided into two subsets stratified according to benign or malignant status, to obtain a training set (70%) and a test set (30%). To avoid bias related to the random split, 100 iterations were performed to create different training and test sets, identified using random seeds.

To assess reproducibility of features between the two readers, the intraclass correlation coefficient (ICC) was calculated (IRR package version 0.84.1). Pairwise ICCs (estimated based on a single-rating, an absolute-agreement, and two-way random-effects model) <0.8 were considered non-reproducible and were excluded. A Spearman correlation analysis was then used to exclude redundant features. Highly correlated features with coefficients >0.9 or <−0.9 were considered redundant and only one was retained. This was made 100 times, once per seed.

**Fig 2:**
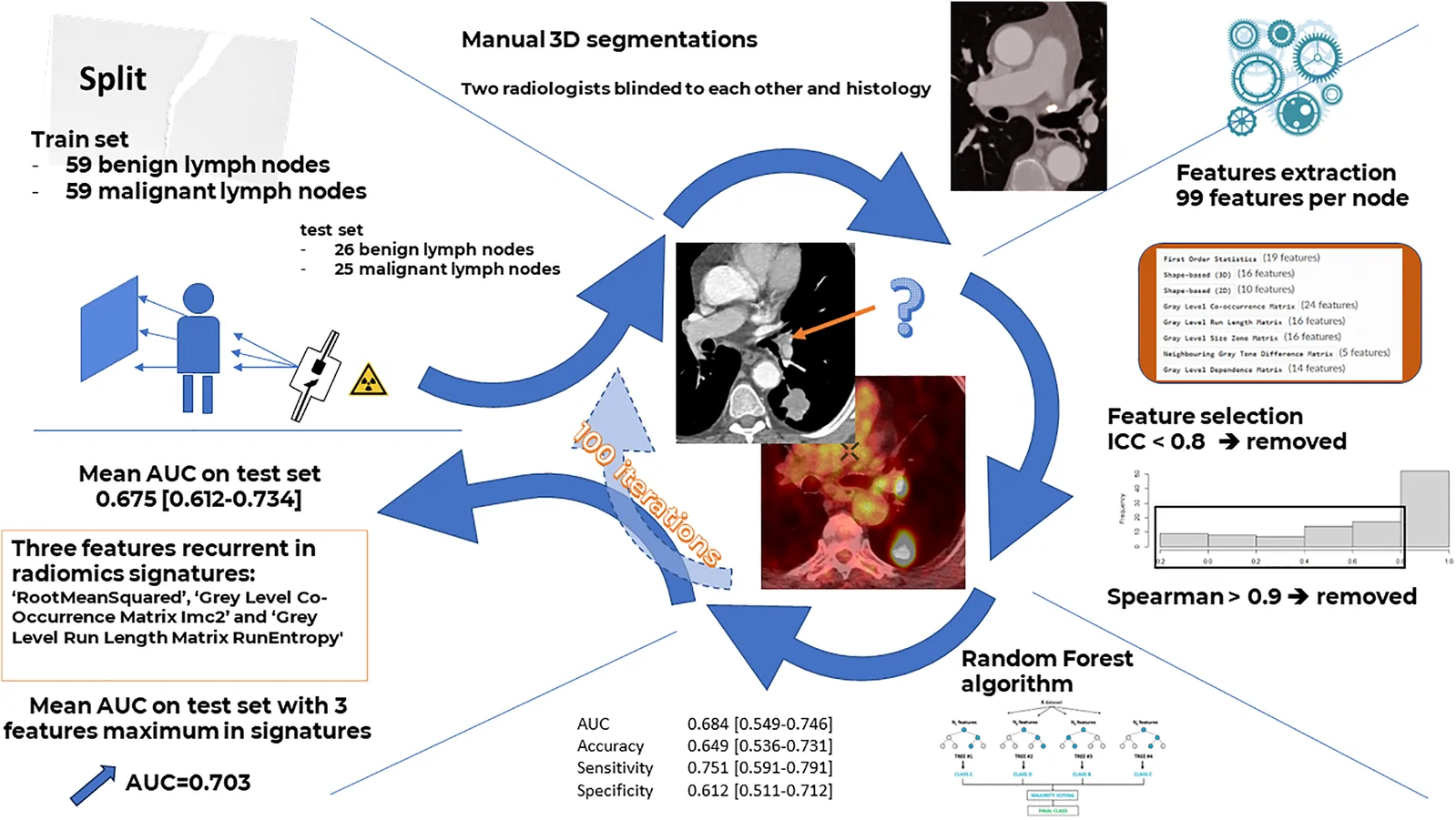
Study Pipeline: Creation of a radiomic signature to distinguish benign from malignant lymph nodes using a random forest classifier with hyperparameter tuning via grid search cross-validation.

To further reduce the number of features, two selection methods were tested using a sequential step-forward feature selection using the bagging classifier based on the AUC obtained on the training set. First, the optimal number of features was automatically selected, based on the best AUC across the 100 iterations (for each seed). Second, the number of parameters has been chosen according to the most recurrent features in the radiomic signature according to the previous method. A radiomic signature was generated to distinguish malignant from benign lymph nodes using a random forest and a XGboost algorithm with hyperparameter tuning via grid search cross-validation (Fig 2). The Forest model codes are available in the appendix (Appendix 1).

A second XGBoost model was used to confirm the results.

### Statistical analysis

Continuous variables with normal distribution are reported as means and standard deviations (95% CI computed using bootstraps with 1000 repetitions). Categorical variables are reported as numbers with percentages.

Statistical analyses were performed with R (version 4.1.1). Python (version 3.8.8) was used to extract features with Pyradiomics package [18] (version 3.0.1), then the Mlxtend package (version 0.24.1) for preprocessing and feature selection, and Scikit-Learn package (version 0.24.1) for machine learning and performance evaluation.

## Results

### Patient and lymph node characteristics

**Fig 3:**
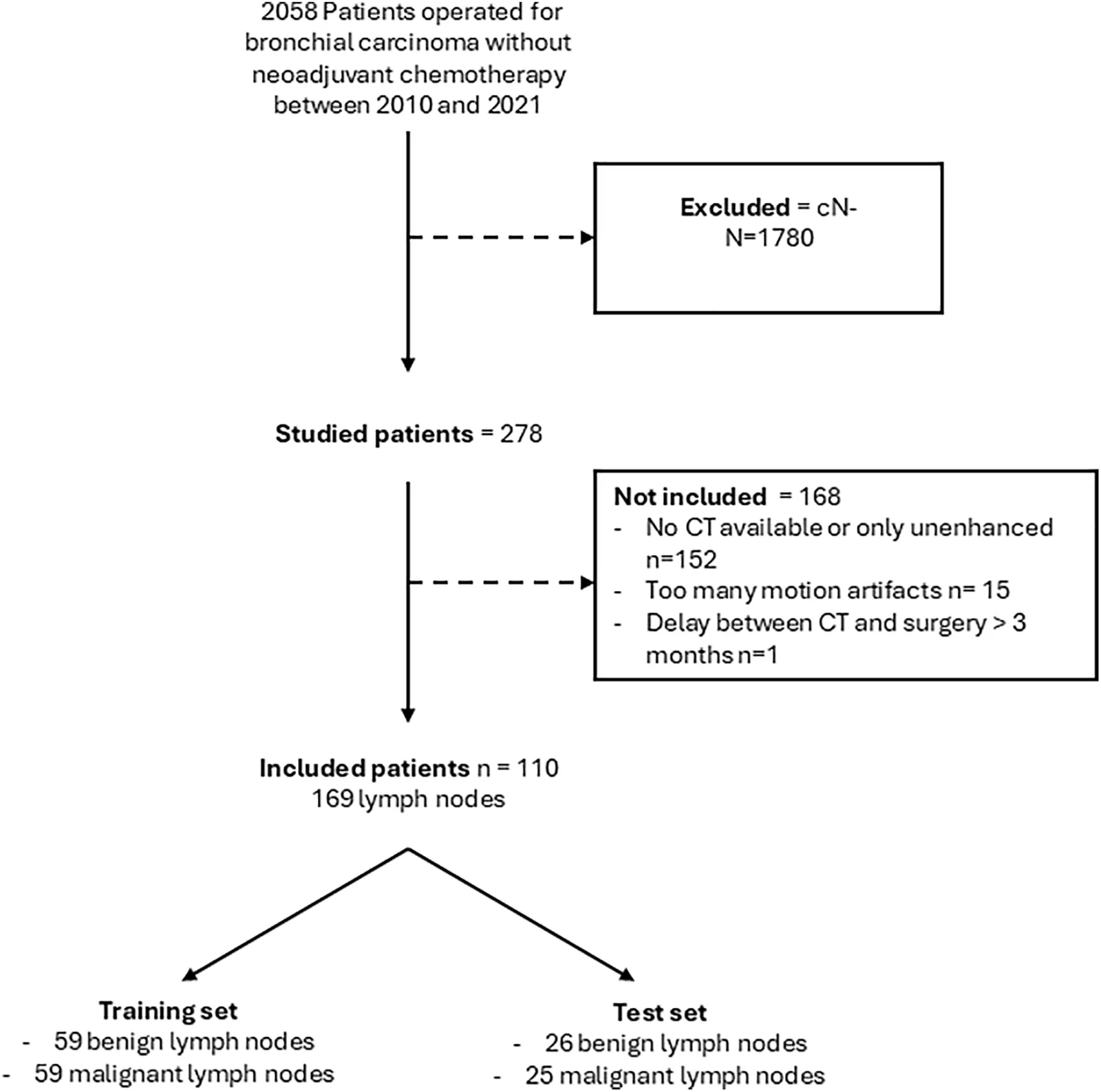
Flow chart of patient inclusion in the training set and test set.

During the study period, 2,058 patients were operated on with lymph node dissection for NSCLC without neoadjuvant chemotherapy. A total of 278 patients met the inclusion criteria (76 pN0 and 202 pN+), and 168 patients were excluded (Fig 3). A total of 110 patients were analyzed (82 [74.5%] men, median age: 65 years [IQR = 12]). They involved 169 lymph nodes: 76 pN+ patients had 115 lymph nodes harvested, of which 86 (74.8%) were malignant; and 34 pN0 patients had 54 benign lymph nodes. Sixty-seven (60.9%) patients had only one cN+ lymph node, 27 (24.5%) had two, and 16 (14.5%) had three cN+ lymph nodes.

The median attenuation values of lymph nodes on CT were comparable between benign (67 [37.5] UH) and malignant (67.5 [33.75] HU) groups.

Regarding primary tumor histology, adenocarcinoma was the most frequent subtype (59% of cases overall), followed by squamous cell carcinoma (36%). Other histological types were less common, including carcinoid tumors (3%), large cell carcinoma (1%), and small cell carcinoma (1%).

Tumors were relatively evenly distributed between the right and left lungs. The right upper lobe (33%) and left upper lobe (25%) were the most frequent locations. Patients’ demographics are presented in Table 1.

**Table 1:** Population characteristics.

	Benign node	Malignant node	Global population
**Patients**	57 (52)	53 (48)	110
Gender			
Male	49 (86)	33 (62)	82 (75)
Female	8 (14)	20 (38)	28 (25)
Age (years)	68 [11]	63 [14]	65 [12]
**CT**			
Delay between CT and surgery (days)	28 [44]	27 [43]	28 [44]
Injection time			
Arterial (35s)	46 (81)	40 (75)	86 (78)
Portal (70s)	11 (19)	13 (25)	24 (22)
**Primary Tumor**			
Size (mm)	38 [42]	26 [17]	32 [24]
Lobe			
RUL	22 (39)	14 (26)	36 (33)
RML	1 (2)	1 (2)	2 (2)
RLL	15 (26)	11 (21)	26 (24)
LUL	12 (21)	16 (30)	28 (25)
LLL	7 (12)	11 (21)	18 (16)
Histology			
Adenocarcinoma	30 (52.5)	35 (66)	65 (59)
Squamous cell carcinoma	24 (42)	16 (30)	40 (36)
Carcinoïd tumor	2 (3.5)	1 (2)	3 (3)
Large cell carcinoma	0 0)	1 (2)	1 (1)
Small cell carcinoma	1 (2)	0 (0)	1 (1)
**Nodes**			
Number	83 (49)	86 (51)	169
Short axis (mm)	11 [3]	13 [6]	12 [5]
2D ROI (HU)	67 [37.5]	67.5 [33.75]	67 [35]
SUV_max_	3.7 [1.05]	6.3 [4.55]	4.2 [3.6]
Anatomic situation			
2	3 (3.6)	2 (2.3)	5 (2.9)
3	1 (1.2)	3 (3.5)	4 (2.4)
4	17 (20.5)	10 (11.6)	27 (16)
5	6 (7.2)	4 (4.7)	10 (5.9)
6	2 (2.4)	0 (0)	2 (1.2)
7	12 (14.5)	11 (12.8)	23 (13.6)
8	0 (0)	1 (1.2)	1 (0.6)
10	17 (20.5)	21 (24.4)	38 (22.5)
11	17 (20.5)	26 (30.2)	43 (25.4)
12	7 (8.4)	7 (8.1)	14 (8.3)
13	1 (1.2)	1 (1.2)	2 (1.2)

Continuous variables are presented as median and interquartile range.

Categorical variables are presented as numbers and percentages.

### Development and performance of the Radiomic Signature

The random forest model was trained and tested 100 times on 100 different splits of the population, after feature reduction based on the deletion of non-reproducible and redundant features. With a bagging classifier used to select features, the diagnostic performance showed a mean AUC of 0.67 [0.61–0.73], accuracy 0.62 [0.56–0.68], sensitivity 0.63 [0.47–0.79] and specificity 0.60 [0.46–0.74].

**Fig 4:**
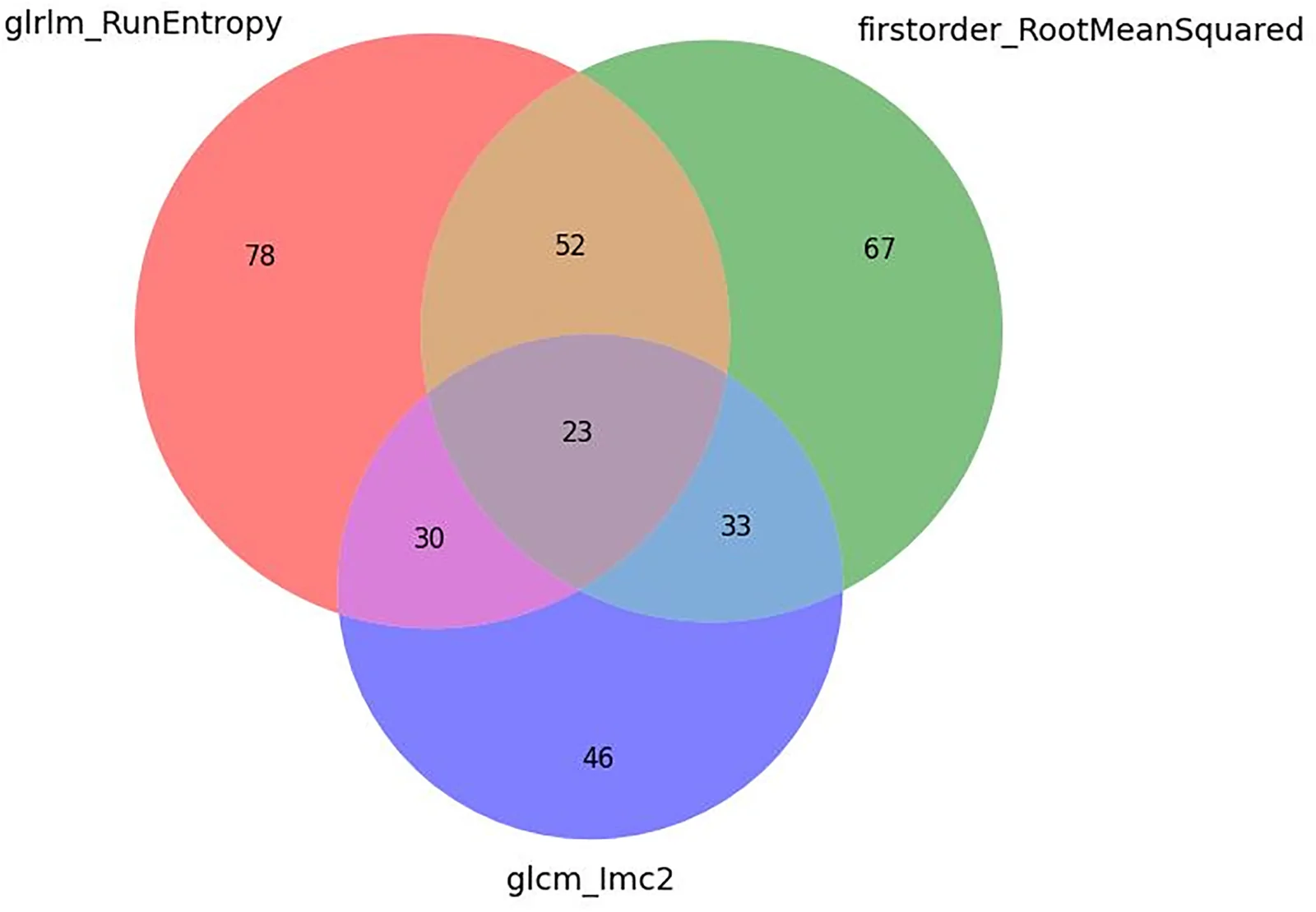
Venn diagram of the top three radiomic features across 100-fold train-test splits (random forest).

Three features were highly represented in the different iterations, present in more than 46% of cases: one first-order feature (RootMeanSquared) and two second-order texture features (Grey Level Co-Occurrence Matrix Imc2 and Grey Level Run Length Matrix RunEntropy). Their interactions are summarized in the Venn diagram (Fig 4).

**Fig 5:**
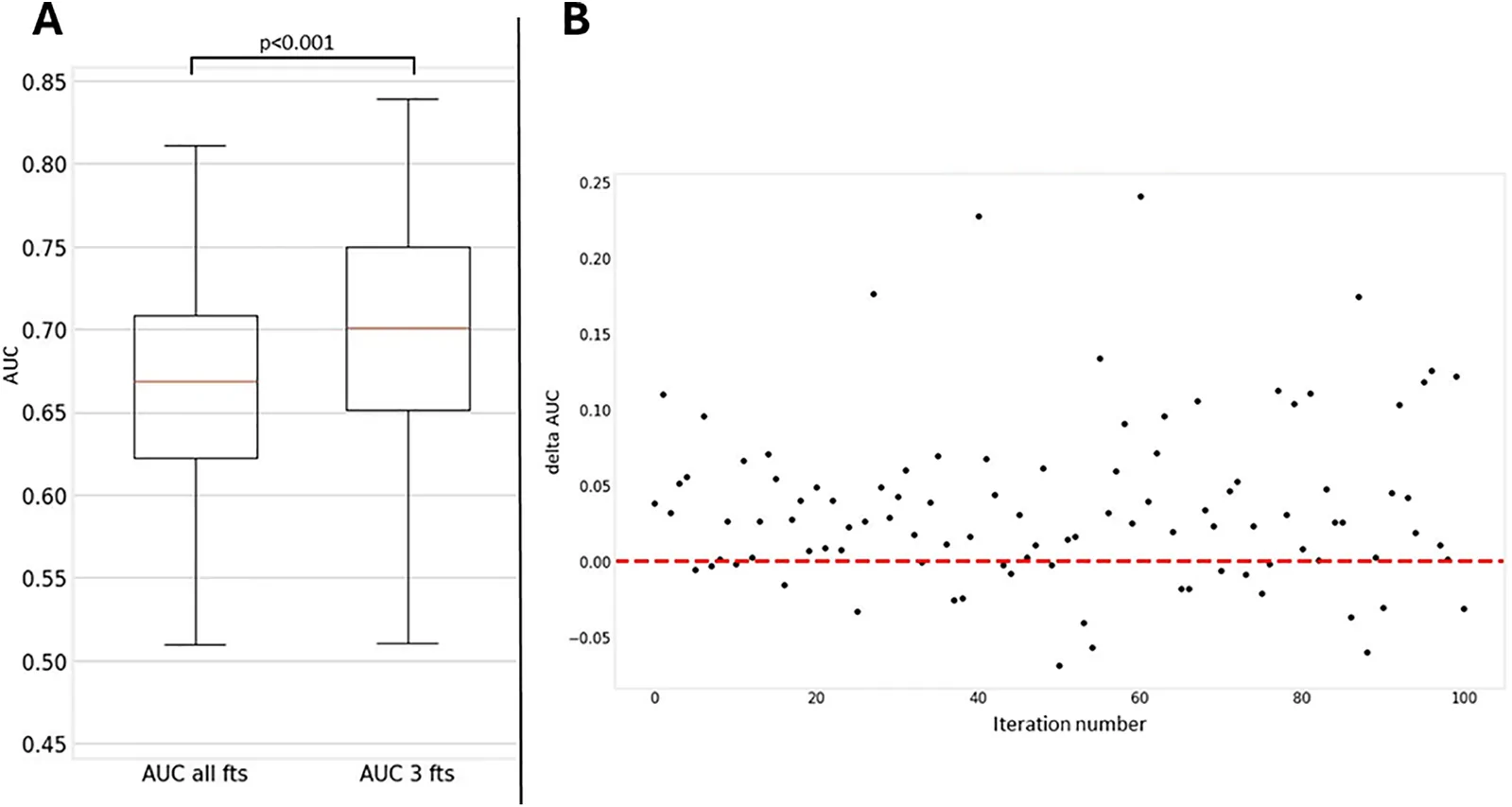
Difference in AUC values measured between all features selection and 3-features selection. A., box plot of the AUC value. B. Bland & Altman like graph illustrating to difference for each iteration.

These radiomic features were selected to establish the radiomic signature. This radiomics model showed a significantly better discriminative performance than the radiomic signature including all selected features by the classifier and yielded higher AUC, accuracy, and sensitivity 0.703, 0.644, 0.742 respectively (Fig 5). Although specificity decreased from 0.60 to 0.54.

**Fig 6:**
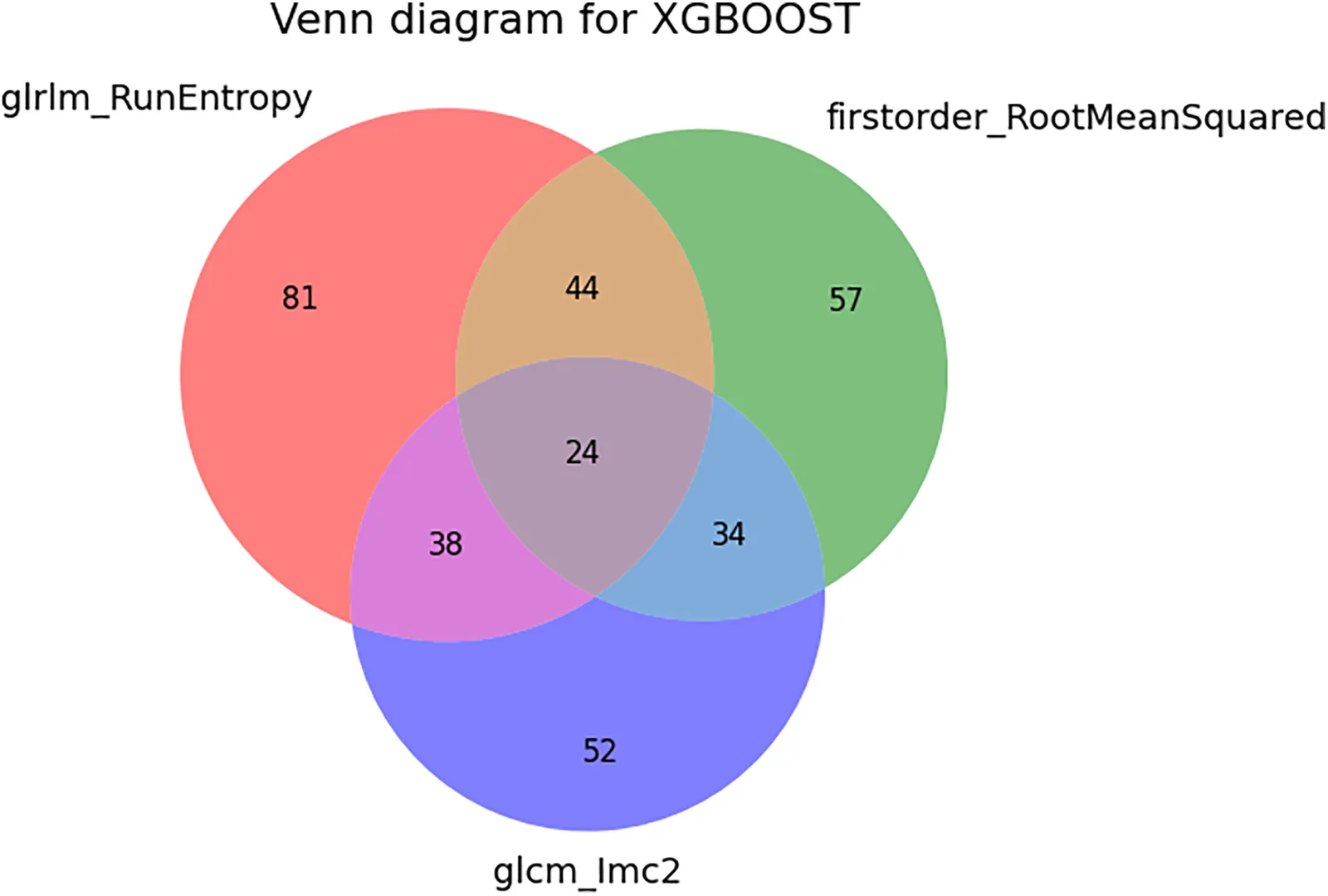
Venn diagram of the top three radiomic features across 100-fold train-test splits (random XGBoost).

**Fig 7:**
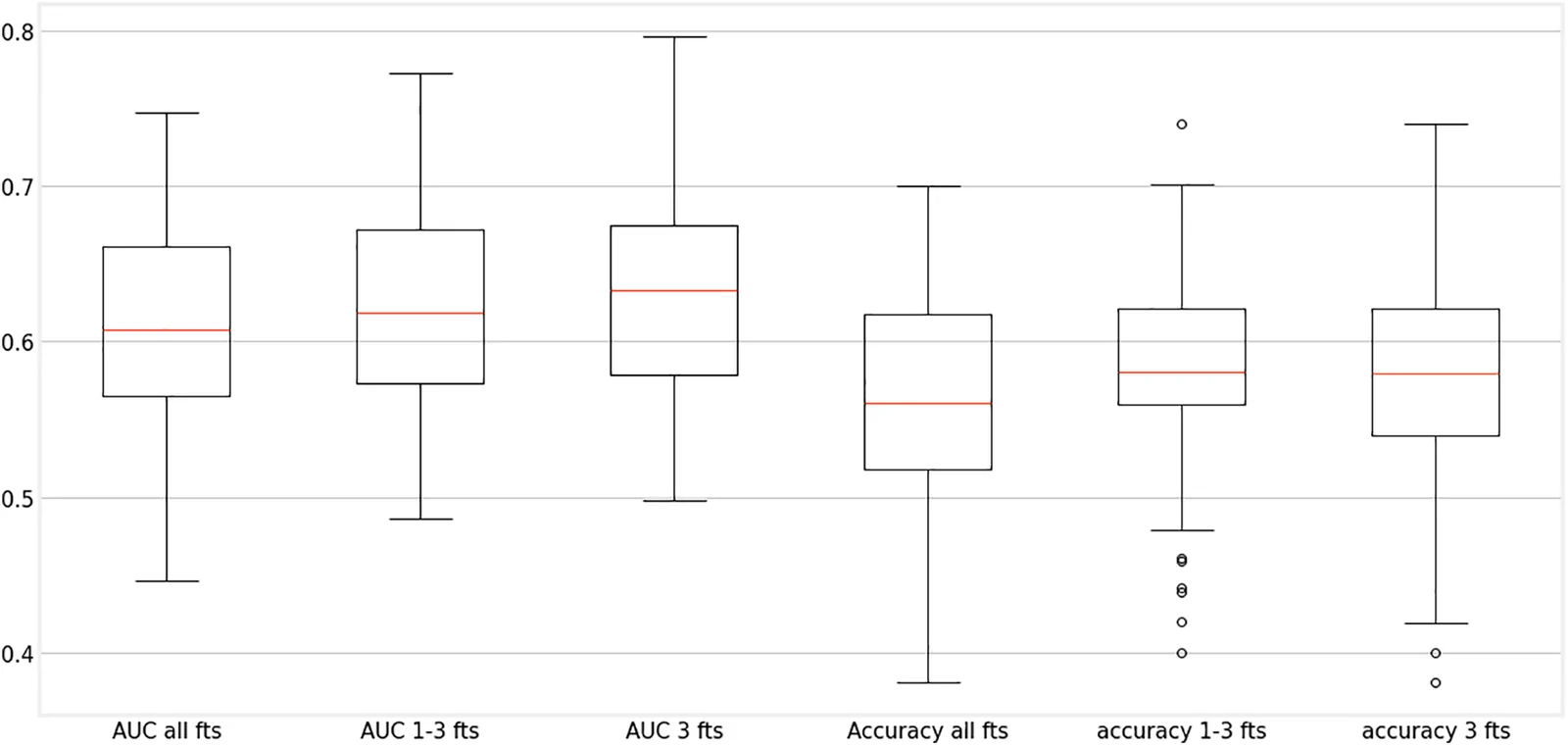
Difference in AUC and accurancy values measured between all features selection and 3-features selection. A., box plot of the AUC and accurancy values, in XGBoost model.

The experiment was repeated with another model: XGBoost model (Fig 6). As with the Forest model, the use of three radiomic features showed better results than with a bagging classifier used to select features in terms of AUC (0.59 vs. 0.63 for three features) and sensitivity (0.49 vs. 0.54 for three features) (Fig 7). However specificity (0.71 to 0.63) and accuracy ((0.59 vs. 0.58) decreased.

## Discussion

This retrospective study on 110 patients with 169 cN+ lymph nodes aimed to predict the occurrence of pN+ or inflammatory nodes according to the radiomic signature of hilar or mediastinal lymph nodes. The model reached an AUC of 0.67 [0.61–0.73] on a test set. The model was trained 100 times on 100 different splits of the sample, and three features of heterogeneity (‘RootMeanSquared’, ‘Grey Level Co-Occurrence Matrix Imc2’, and ‘Grey Level Run Length Matrix RunEntropy’) were recurrent in the radiomic signature. To the best of our knowledge, this is the first study to perform this goal using 3D segmentation of the node in a Caucasian population.

In the field of radiomics analysis for lymph node evaluation in NSCLC, prior research has explored the diagnostic potential of radiomics models by segmenting the lymph nodes rather than the tumor, as done in the present study. A study by Dong et al. [14] presented results based on contrast-enhanced chest CT, reporting a model with an AUC of 0.803 on the test set, although using 2D segmentations. Like in our study, they found that Grey Level Co-Occurrence Matrix feature was an important radiomics category for predicting lymph node malignancy, including three such features in their model. These features reflected the combined probability of specific pixel pairs exhibiting particular gray-level values. Another study [19] developed models based on different CT imaging phases using delta radiomics (unenhanced, arterial, and portal) to diagnose mediastinal lymph node metastasis, achieving strong discriminatory performance (AUC > 0.8) but again based on 2D segmentation, which may be influenced by measurement variability. In both studies, only a single split of the cohort was performed. A third earlier study of 2015 [20], prior to the emergence of radiomics, used textural analysis to differentiate malignant and benign lymph nodes, achieving a good AUC of 0.83 with high specificity (97%) and moderate sensitivity (53%) with 3D ROIs. They found that the mean HU of 3D ROIs was higher for malignant lymph nodes than for benign ones (75.3 vs 55.6, respectively; p < 0.001). Interestingly, the size of the node was not statistically different between benign or malignant groups. These three studies were conducted using CT scans from only one vendor. In contrast, the AUC in our study was lower, but our strengths included: the use of 3D segmentations, imaging data from multiple CT vendors, and a 100-fold training process to avoid random association with a single cohort split [21].

Overall, previous studies have reported promising diagnostic performance of CT-based radiomics for the assessment of mediastinal lymph nodes in non-small cell lung cancer; however, most relied on 2D segmentations, single-cohort divisions, and data from a single provider, which limited their reproducibility and generalizability. These limitations underscore the need for more robust approaches incorporating 3D segmentation, data from multiple providers, and repeated validation strategies, as proposed in the present study.A recent investigation [22] assessed CT texture analysis in addition to size and introduced the Node-RADS classification for discriminating lymph node malignancy. This is a 5-point scoring system that considers the configuration of the node (homogeneous, heterogeneous, necrotic). There was a crucial need for this kind of score. Very interestingly, the three repetitive features found in the 100-fold were linked to heterogeneity in the grey-level matrix within the segmentation, and none were related to shape, supporting the hypothesis that lymph node heterogeneity is a strong predictor of malignancy, along with size criterion. This has already been published but is still not widely accepted in the literature and scientific guidelines [23,24].

In addition to studies focusing on lymph node analysis, other research has delved into tumour segmentation instead of node segmentation to predict the risk of cN+ status. One study by Botta et al. [25] aimed to evaluate the association between radiomic and clinical features with lymph node status and overall survival, involving 270 patients. The results indicated that the radiomics model did not outperform single clinical features in predicting positive lymph nodes. Importantly, the choice of CT reconstruction algorithms also influenced model performance.

Studies that seek to segment the tumor are more concerned with predicting the patient’s postoperative category according to the characteristics of the main lesion or the probability of having a positive node, whatever its position in the mediastinum. They do not address the problem of false positives resulting from CT and PET scans, but rather occult lymph node metastatic disease [26]. Other non-radiomics morphological studies have already been published on this subject, with prediction scores leading to high diagnostic performance, as in the study by Guinde et al. with an AUC 0.85 [0.80–0.90] [27]. The difference with the study presented here was to extract from the radiomics the semantic imaging features that seemed most interesting and relevant for the model to classify lymph nodes, and to envisage future research to define simple imaging criteria for everyday practice.

In the quest for accurate clinical staging of mediastinal lymph nodes in lung cancer patients, MRI could have a role that deserves further exploration. A review [28] evaluated the diagnostic performance of diffusion-weighted magnetic resonance imaging (DWI) and PET/CT in detecting mediastinal nodal metastasis. This analysis incorporated data from 43 studies. For PET/CT, the pooled sensitivity and specificity were 0.65 and 0.93, respectively, while DWI demonstrated a higher pooled sensitivity of 0.72 and specificity of 0.97. The positive likelihood ratio for DWI was 13.15, indicating a strong diagnostic capability, while PET/CT also exhibited a notable positive likelihood ratio of 8.46. However, potential sources of heterogeneity existed, with study design and patient enrolment impacting the threshold variations.

Improvements in the diagnostic performance of radiomics imaging for cN+ could lead to a reduction in the number of invasive procedures currently required for mediastinal staging of these patients. The morbidity and mortality of these procedures are low, but they do exist, lengthening the time it takes to treat patients. Since NSCLC is a rapidly progressive cancer, and delay in treatment represents a loss of chance for the patient [29].

As has recently been pointed out, radiomics could also facilitate the establishment of standardized imaging biobanks that integrate quantitative imaging, clinical, and histopathological data, thereby contributing to precision medicine in oncology [30].

Our study has several limitations. It is a retrospective single-center study without an external validation set. The sample size is relatively small. No clinical factors were integrated into the model, which might further improve performance. Additionally, anatomically linking the dissected nodes described in histopathological reports to those seen on the enhanced chest CT could be challenging.

## Conclusion

This study demonstrates that radiomic features extracted from enhanced thoracic CT scans can predict the malignant status of cN+ mediastinal lymph nodes in NSCLC patients, with an AUC of 0.703. The radiomics model, based on 3D lymph node segmentations, shows that three features are recurrent in radiomic signatures: “RootMeanSquared”, “Grey Level Co-Occurrence Matrix Imc2”, and “Grey Level Run Length Matrix RunEntropy”. These are heterogeneity features, which support the idea that a node heterogeneity criterion is needed in addition to short-axis diameter, which is currently the only diagnostic criterion. Further validation studies are warranted.

### Key points

Radiomic features of thoracic CT can predict lymph node malignancy in NSCLC.

RootMeanSquared, GLCM Imc2, and GLRLM RunEntropy are key heterogeneity markers.

Radiomics suggests node heterogeneity should complement size in malignancy assessment.

## Supporting information

S1 Table Specifications of the scanners used. (DOCX)

S1 Appendix Forest Model Codes. (IPYNB)

## Abbreviations

NSCLC: Non-small cell lung cancer
AUC: Areas Under the Curve
CT: Computed Tomography
PET: Positron Emission Tomography
