# Supplementary material for: CT radiomics-histopathological correlation for mediastinal lymph node staging in non-small cell lung cancer
Source: PLoS One. 2026 Aug 31;21(8):e0355650. doi: 10.1371/journal.pone.0355650 (PMC13528979; doi:10.1371/journal.pone.0355650)
Supplement: Supplementary file 1 [file pone.0355650.s001.docx]

Supplementary data

Table e1 :

| **N°** | **Brand** | **Model** | **kV** | **mA** | **Kernel** | **Slice**  **Thickness (mm)** | **Injection Delay (s)** | **Reconstruction Diameter (mm)** | **Spacing Between Slices (mm)** | **Contrast Agent**  **And concentration (mG/mL)** | **Contrast Bolus Volume (mL)** | **Contrast Flow Rate (mL/s)** |
| --- | --- | --- | --- | --- | --- | --- | --- | --- | --- | --- | --- | --- |
| 1 | SIEMENS | SOMATOM Definition AS | 120 | 186 | B30f | 1.5 | 70 | 426 |  |  |  |  |
| 2 | SIEMENS | SOMATOM Definition AS | 100 | 544 | ['I30f', '3'] | 2 | 35 | 414 |  | Iopamiron  370 |  |  |
| 3 | GE | Optima CT660 | 120 | 185 | STANDARD | 1.25 | 70 | 432 | 1.25 |  |  |  |
| 4 | GE | LightSpeed VCT | 120 | 151 | SOFT | 1.25 |  | 500 | 1 |  |  |  |
| 5 | GE | Discovery CT750 HD | 120 | 458 | STANDARD | 1.25 |  | 384 | 5 |  |  |  |
| 6 | Philips | Brilliance 64 | 120 | 442 | C | 2 |  | 464 | 1 |  |  |  |
| 7 | SIEMENS | Sensation Cardiac 64 | 120 | 562 | B30f | 1.5 | 70 | 400 |  |  | 100 | 3 |
| 8 | SIEMENS | Sensation Cardiac 64 | 120 | 387 | B30f | 1.5 |  | 368 |  |  |  |  |
| 9 | SIEMENS | Sensation Cardiac 64 | 120 | 229 | B30f | 1.5 | 70 | 384 |  |  | 100 | 3 |
| 10 | GE | Optima CT660 | 120 | 286 | STANDARD | 1.25 | 70 | 358 | 1.25 |  |  |  |
| 11 | SIEMENS | Sensation Cardiac 64 | 120 | 75 | B30f | 1.5 | 35 | 359 |  |  | 100 | 3 |
| 12 | SIEMENS | Sensation Cardiac 64 | 120 | 521 | B30f | 1.5 | 70 | 368 |  |  |  |  |
| 13 | SIEMENS | Sensation Cardiac 64 | 120 | 576 | B30f | 1.5 | 70 | 353 |  |  |  |  |
| 14 | SIEMENS | Sensation Cardiac 64 | 120 | 579 | B30f | 1.5 | 70 | 412 |  |  |  |  |
| 15 | SIEMENS | Sensation 64 | 120 | 414 | B30f | 1.5 | 70 | 411 |  |  | 100 | 3 |
| 16 | GE | LightSpeed VCT | 120 | 533 | SOFT | 1.25 | 70 | 435 | 1 | OMNIPAQUE  350 |  |  |
| 17 | GE | Optima CT660 | 100 | 479 | STANDARD | 1.25 | 70 | 380 | 0.625 |  |  |  |
| 18 | SIEMENS | SOMATOM Definition | 120 | 641 | B26f | 0.75 | 35 | 381 |  |  | 75 | 4,5 |
| 19 | GE | LightSpeed VCT | 120 | 437 | SOFT | 1.25 | 70 | 478 | 1 |  |  |  |
| 20 | GE | Optima CT660 | 100 | 254 | STANDARD | 1.25 |  | 360 | 1.25 |  |  |  |
| 21 | GE | Optima CT660 | 120 | 409 | STANDARD | 1.25 | 70 | 434 | 1.25 |  |  |  |
| 22 | GE | Optima CT660 | 120 | 152 | STANDARD | 1.25 | 35 | 360 | 1.25 |  |  |  |
| 23 | GE | Discovery CT750 HD | 100 | 480 | STANDARD | 0.625 | 70 | 400 | 0.625 |  |  |  |
| 24 | SIEMENS | Sensation Cardiac 64 | 120 | 580 | B30f | 1.5 | 70 | 384 |  |  |  |  |
| 25 | SIEMENS | Sensation Cardiac 64 | 120 | 577 | B30f | 1.5 | 70 | 416 |  |  |  |  |
| 26 | SIEMENS | Sensation Cardiac 64 | 120 | 395 | B30f | 1.5 | 70 | 363 |  |  |  |  |
| 27 | GE | Optima CT660 | 120 | 498 | STANDARD | 1.25 | 70 | 462 | 1.25 |  |  |  |
| 28 | GE | Optima CT660 | 120 | 499 | STANDARD | 1.25 | 70 | 500 | 1.25 |  |  |  |
| 29 | GE | LightSpeed VCT | 120 | 192 | SOFT | 1.25 | 70 | 400 | 1 |  |  |  |
| 30 | GE | Optima CT660 | 120 | 200 | STANDARD | 1.25 | 70 | 400 | 1.25 |  |  |  |
| 31 | GE | Revolution EVO | 120 | 275 | STANDARD | 1.25 |  | 418 | 1.25 |  |  |  |
| 32 | GE | Optima CT660 | 120 | 362 | STANDARD | 1.25 | 70 | 497 | 1.25 |  |  |  |
| 33 | GE | Optima CT660 | 120 | 93 | STANDARD | 1.25 | 70 | 396 | 1.25 |  |  |  |
| 34 | SIEMENS | Sensation Cardiac 64 | 120 | 575 | B30f | 1.5 | 70 | 415 |  |  |  |  |
| 35 | SIEMENS | Sensation Cardiac 64 | 120 | 554 | B30f | 1.5 | 70 | 418 |  |  |  |  |
| 36 | GE | Discovery CT750 HD | 120 | 253 | STANDARD | 1.25 | 35 | 427 | 1 |  |  |  |
| 37 | GE | Optima CT660 | 120 | 299 | STANDARD | 1.25 | 35 | 500 | 1.25 |  |  |  |
| 38 | GE | Revolution HD | 120 | 167 | STANDARD | 1.25 | 70 | 400 | 0.625 |  |  |  |
| 39 | SIEMENS | SOMATOM Definition | 100 | 549 | B30f | 1 | 70 | 420 |  |  |  |  |
| 40 | SIEMENS | Sensation Cardiac 64 | 120 | 345 | B30f | 1.5 | 35 | 400 |  |  | 100 | 3 |
| 41 | GE | Optima CT660 | 120 | 250 | STANDARD | 1.25 | 70 | 383 | 1.25 |  |  |  |
| 42 | SIEMENS | Sensation Cardiac 64 | 120 | 431 | B30f | 1.5 | 70 | 394 |  |  | 100 | 3 |
| 43 | GE | Discovery CT750 HD | 100 | 282 | STANDARD | 0.625 | 70 | 338 | 0.625 |  |  |  |
| 44 | GE | Optima CT660 | 120 | 181 | STANDARD | 1.25 | 35 | 463 | 1.25 |  |  |  |
| 45 | SIEMENS | SOMATOM Definition | 120 | 87 | B20f | 1 | 70 | 500 |  |  |  |  |
| 46 | GE | Discovery CT750 HD | 100 | 290 | STANDARD | 0.625 | 70 | 339 | 0.625 |  |  |  |
| 47 | GE | Discovery CT750 HD | 120 | 234 | STANDARD | 1.25 | 35 | 452 | 1 |  |  |  |
| 48 | GE | Revolution HD | 120 | 100 | STANDARD | 1.25 | 70 | 377 | 0.625 |  |  |  |
| 49 | SIEMENS | Sensation Cardiac 64 | 120 | 578 | B30f | 1.5 | 70 | 368 |  |  |  |  |
| 50 | GE | Optima CT660 | 120 | 178 | STANDARD | 1.25 | 70 | 500 | 1.25 |  |  |  |
| 51 | GE | Revolution HD | 120 | 454 | STANDARD | 1.25 |  | 500 | 1.25 |  |  |  |
| 52 | GE | Optima CT660 | 120 | 395 | STANDARD | 1.25 | 70 | 360 | 1.25 |  |  |  |
| 53 | GE | Optima CT660 | 120 | 363 | STANDARD | 1.25 | 35 | 415 | 1.25 |  |  |  |
| 54 | GE | Revolution HD | 120 | 144 | STANDARD | 1.25 | 70 | 375 | 0.625 |  |  |  |
| 55 | GE | Optima CT660 | 120 | 200 | STANDARD | 1.25 |  | 398 | 1.25 |  |  |  |
| 56 | SIEMENS | SOMATOM Scope | 130 | 180 | ['I30s', '2'] | 1.5 | 70 | 400 |  |  |  |  |
| 57 | SIEMENS | SOMATOM Definition | 120 | 383 | B30f | 1 | 70 | 444 |  |  |  |  |
| 58 | GE | Optima CT660 | 120 | 183 | STANDARD | 1.25 |  | 421 | 1.25 |  |  |  |
| 59 | GE | Revolution HD | 120 | 163 | STANDARD | 1.25 | 70 | 350 | 0.625 |  |  |  |
| 60 | GE | Optima CT660 | 120 | 295 | STANDARD | 1.25 | 35 | 444 | 1.25 |  |  |  |
| 61 | SIEMENS | Sensation Cardiac 64 | 120 | 214 | B30f | 1.5 | 70 | 444 |  |  | 100 | 3 |
| 62 | GE | Revolution HD | 120 | 334 | STANDARD | 1.25 | 35 | 476 | 1.25 |  |  |  |
| 63 | GE | Revolution HD | 120 | 184 | STANDARD | 1.25 |  | 500 | 1.25 |  |  |  |
| 64 | GE | Optima CT660 | 120 | 183 | STANDARD | 1.25 | 70 | 373 | 1.25 |  |  |  |
| 65 | GE | Optima CT540 | 120 | 333 | STANDARD | 1.25 |  | 376 | 1.25 |  |  |  |
| 66 | GE | Revolution EVO | 120 | 195 | STANDARD | 0.625 | 70 | 401 | 0.625 |  |  |  |
| 67 | TOSHIBA | Aquilion PRIME | 120 | 100 | FC17 | 1 |  | 400,39 |  |  |  |  |
| 68 | GE | LightSpeed VCT | 120 | 355 | SOFT | 1.25 | 70 | 477 | 1 | ULTRAVIST  370 |  |  |
| 69 | SIEMENS | SOMATOM Definition | 120 | 186 | B20f | 1 | 35 | 434 |  |  |  |  |
| 70 | SIEMENS | Sensation 64 | 120 | 576 | B30f | 1.5 |  | 378 |  |  |  |  |
| 71 | GE | Revolution HD | 120 | 337 | STANDARD | 1.25 | 35 | 456 | 1.25 |  |  |  |
| 72 | SIEMENS | Sensation 64 | 120 | 576 | B30f | 1.5 | 70 | 355 |  |  |  |  |
| 73 | SIEMENS | Sensation 64 | 120 | 480 | B30f | 1.5 | 35 | 376 |  |  | 100 | 3 |
| 74 | GE | Optima CT660 | 120 | 485 | STANDARD | 1.25 | 70 | 383 | 1.25 |  |  |  |
| 75 | SIEMENS | Sensation 64 | 120 | 379 | B30f | 1.5 |  | 280 |  |  |  |  |
| 76 | GE | LightSpeed VCT | 120 | 496 | STANDARD | 1.25 |  | 492 | 1 |  |  |  |
| 77 | GE | Discovery CT750 HD | 100 | 200 | STANDARD | 0.625 | 70 | 407 | 0.625 |  |  |  |
| 78 | GE | LightSpeed VCT | 100 | 546 | SOFT | 1.25 | 35 | 378 | 1 |  |  |  |
| 79 | GE | LightSpeed VCT | 120 | 458 | STANDARD | 1.25 | 70 | 418 | 1 | XENETIX  350 |  |  |
| 80 | GE | Discovery CT750 HD | 120 | 338 | STANDARD | 1.25 |  | 422 | 1 | ultravist  370 |  |  |
| 81 | GE | LightSpeed VCT | 100 | 365 | STANDARD | 1.25 |  | 346 | 1 |  |  |  |
| 82 | GE | LightSpeed VCT | 120 | 359 | STANDARD | 1.25 |  | 417 | 1 |  |  |  |
| 83 | GE | Optima CT660 | 120 | 366 | STANDARD | 1.25 | 70 | 402 | 1.25 |  |  |  |
| 84 | GE | LightSpeed VCT | 120 | 358 | STANDARD | 1.25 |  | 434 | 1 |  |  |  |
| 85 | SIEMENS | Sensation Cardiac 64 | 120 | 303 | B30f | 1.5 | 35 | 383 |  |  | 100 | 3 |
| 86 | SIEMENS | Sensation Cardiac 64 | 120 | 283 | B30f | 1.5 |  | 414 |  |  | 100 | 3 |
| 87 | SIEMENS | Sensation 64 | 120 | 213 | B30f | 1 |  | 380 |  |  |  |  |
| 88 | SIEMENS | Sensation Cardiac 64 | 120 | 572 | B30f | 1.5 | 70 | 433 |  |  | 100 | 3 |
| 89 | GE | Revolution EVO | 100 | 367 | STANDARD | 1.25 | 70 | 360 | 0.625 |  |  |  |
| 90 | GE | LightSpeed VCT | 120 | 496 | STANDARD | 1.25 |  | 418 | 1 | OMNIPAQUE  320 |  |  |
| 91 | SIEMENS | Definition | 100 | 561 | B30f | 1 | 70 | 458 |  |  |  |  |
| 92 | SIEMENS | Sensation 64 | 120 | 498 | B30f | 1.5 |  | 334 |  |  |  |  |
| 93 | GE | Optima CT660 | 120 | 421 | STANDARD | 1.25 | 70 | 398 | 1.25 |  |  |  |
| 94 | SIEMENS | Sensation 64 | 120 | 221 | B30f | 1.5 |  | 393 |  |  | 100 | 3 |
| 95 | SIEMENS | Sensation 64 | 120 | 548 | B30f | 1.5 |  | 429 |  |  | 100 | 3 |
| 96 | SIEMENS | Sensation 64 | 120 | 253 | B30f | 1.5 |  | 450 |  |  | 100 | 3 |
| 97 | GE | LightSpeed VCT | 120 | 411 | SOFT | 1.25 |  | 500 | 1 | XENETIX  350 |  |  |
| 98 | GE | LightSpeed VCT | 120 | 404 | STANDARD | 1.25 |  | 459 | 1 |  |  |  |
| 99 | SIEMENS | Sensation Cardiac 64 | 120 | 573 | B30f | 1.5 |  | 367 |  |  |  |  |
| 100 | GE | Discovery CT750 HD | 120 | 125 | STANDARD | 1.25 |  | 400 | 1.25 |  |  |  |
| 101 | SIEMENS | Sensation Cardiac 64 | 120 | 141 | B30f | 1.5 | 70 | 366 |  |  | 100 | 3 |
| 102 | GE | Revolution EVO | 120 | 465 | STANDARD | 1.25 | 35 | 493 | 1.25 |  |  |  |
| 103 | GE | LightSpeed Pro 32 | 120 | 533 | SOFT | 1.25 | 70 | 420 |  | ultravist  300 |  |  |
| 104 | GE | Optima CT660 | 100 | 430 | STANDARD | 1.25 | 70 | 360 | 1.25 |  |  |  |
| 105 | Hitachi | SCENARIA | 120 | 337 | 83 | 1.25 | 70 | 393 |  |  |  |  |
